# Bimodal distribution of thyroid dysfunction triggered by COVID-19 Infection: An experience from a single endocrine center—a case series and literature review

**DOI:** 10.5339/qmj.2022.39

**Published:** 2022-08-05

**Authors:** Tarik Elhadd, Wajiha Gul, Zeinab Dabbous, Stephen Beer, Mohammed Bashir

**Affiliations:** Endocrine Section, Department of Medicine, Hamad Medical Corporation, Doha, Qatar E-mail: tarikelhadd58@gmail.com

**Keywords:** Hyperthyroidism, Thyroiditis, hypothyroidism, COVID-19, Case Series

## Abstract

Background: COVID-19 infection has been spreading across the globe since the end of 2019, and it continues to cause chronic multi-system sequelae, of which thyroid dysfunction appears to be the major one. We have discussed here 10 cases of thyroid dysfunction after COVID-19 infection.

Methods: Case series report. From October 2020 to July 2021, a series of 10 cases of thyroid dysfunction after COVID-19 infection were recorded and managed in a single outpatient endocrine center in Doha, Qatar.

Cases presentation: We have reported 5 cases of Graves's hyperthyroidism, 2 of chronic primary hypothyroidism (including one with Grave's disease [GD]) who was treated through radioactive iodine (RAI) therapy, one case of subacute thyroiditis, one case with “Sick euthyroid disease,” and one case of central hypothyroidism. Presently, patients with GD are being treated with carbimazole and those with hypothyroidism are being treated with levothyroxine. The remaining patients had recovered with euthyroid.

Conclusion: This is the largest case series reported from a single center to date. The findings of this series indicate a bimodal distribution of thyroid dysfunction in patients with COVID-19 infection. A review of the literature and discussion of potential pathophysiological mechanisms has been presented. We have emphasized the importance of screening for thyroid dysfunction in “post-COVID-19” cases, considering that the prevalence may be underestimated.

## Background

For over 2 years, the world has been battling with the health impact stemming from Severe Acute Respiratory Syndrome Coronavirus 2 (SARS-CoV-2), also known as COVID-19 (coronavirus disease 2019), which was first reported in Wuhan in the winter of 2019/2020. The virus classically presents with respiratory symptoms but could well be asymptomatic or may even present with an atypical presentation with non-pulmonary complications that include neurological disorders, cardiac abnormalities, renal failure, liver disease, rhabdomyolysis, coagulopathy, thrombosis, and endocrine dysfunctions.^1^ SARS-CoV-2 uses angiotensin-converting enzyme-2 (ACE-2) receptor and transmembrane protease serine 2 (TMPRSS2), as well as cathpesin to gain access and infect the human cells.^2^ Infection or entry of this virus triggers a cascade of virus replication and the release of the virus, resulting in cell damage. This process is called *pyroptosis*, an abrupt form of programmed cell death,^3^ with a subsequent cascade of events including the release of an array of cytokines (e.g. interleukins, tumor necrosis factor [TNF], and interferon [IFN]-γ), in addition to intracellular molecules such as adenosine triphosphate (ATP), nucleic acids, and pathogen-associated molecular patterns. The proinflammatory cytokines (such as interlukin [IL]-β, TNF-α, IFN-γ) may lead to immune response hyperactivity and uncontrolled systemic inflammatory response.^3^ Enhanced T-helper (Th1/Th17) immune responses and cytokine pathways involved in severe acute respiratory syndrome (SARS) resemble the immune activation occurring in immune-mediated thyroid diseases.^4^ ACE-2 and transmembrane protease serine 2 (TMPRSS2) expression are extremely high in the thyroid more than that in the lungs.^5^

The uptake by host cells of SARS-COVID-2 is believed to be mediated by other cellular proteases and molecules. One main group of a cellular membrane proteins involved was found to be integrins, and ACE2 has been reported to bind to integrins to modulate the downstream signal transduction. Thyroxine (T4) regulates the expression of genes for the monomeric protein that constitutes integrins.^5^ Thyroid hormones regulate genes for the monomeric protein that constitute integrins and are believed to promote the internalization of integrins.^7,8^ Notably, olfactory receptors (ORs) are co-expressed with ACE2 and TMPRSS2. ORs are widely expressed in the thyroid.^9^ Impairment of the cellular function at the neuroepithelium of the olfactory bulb constitutes the molecular mechanism that underlies the anosmia observed in COVID-19 victims.^10^ The damage to ORs is postulated to contribute to the impairment of other organs expressing OR, not excluding the thyroid.^9^

Viruses have been well recognized to trigger thyroid autoimmunity.^11^ Thyroid disease in the context of COVID-19 infection was demonstrated in a recent systematic review and meta-analysis was associated with a poor outcome.^12^ However, the relationship between the two has been described as bidirectional.^13^ In this short report, we have presented 10 cases of thyroid dysfunction associated with COVID-19 infection that was diagnosed and managed at a single endocrine center in Qatar. We have also reviewed the relevant literature to support the case report.

## Case Presentations

• ***Case 1:*** A 49-year-old Sudanese gentleman with a history of bronchial asthma, hypertension, prediabetes, and gout tested positive for COVID-19 on November 24, 2020. He underwent polymerase chain reaction testing as mandatory screening, which was performed upon arrival in Qatar as a part of the preventive measures taken to curb the spread of coronavirus. Upon questioning, he provided a history of headache and fever over the preceding few days along with multiple episodes of vomiting and diarrhea (around 4 times a day). The patient denied any cough, shortness of breath, or sore throat. He was diagnosed with COVID-19 pneumonia and was started on the anti-retroviral favipiravir and transferred to a quarantine facility. No thyroid function tests (TFTs) were conducted during this time. He undertook a TFT in May 2019, which was normal. He subsequently made an uneventful recovery from COVID-19. Two months later, the patient undertook a general checkup to complain of a slight increase in body weight. Here, his TFT revealed a thyroid-stimulating hormone (TSH) value of 33.49 mu/L (normal range reference [NRR] 0.4–4.5 mu/L), and a free tbl4 value of 8 pmol/L (NRR 8–18 pmol/L). Repeat TFTs conducted 6 weeks later revealed a TSH value of 34.22 mu/L and the tbl4 value further dropped to 7.4 pmol/L. At this point, he was referred to our endocrine clinic.
  When reviewed at our clinic, he denied any history of tiredness, fatigue, sleepiness, or other symptoms of hypothyroidism other than weight gain. He had no family history of goiter or thyroid dysfunction. He was not on any medication that may have caused perturbation of the thyroid functions. On examination, his vital signs were normal. He was clinically euthyroid, with no neck swelling and an unremarkable physical examination. The test results showed TSH 49 mU/L and FT 11 pmol/L. Antithyroid peroxidase antibodies (anti-TPO Ab) were 423 IU/mL (normal < 30) and TSH receptor antibodies TRAb was negative. Neck ultrasound (US) showed heterogeneous thyroid with minimally increased vascularity and a small hyperechoic nodule measuring 5 x 4 mm. The thyroid uptake scan revealed normal morphology and function, except for a cold nodule in the postero-inferior part of the right lobe.
  After a discussion with the patient, it was decided to adopt the wait-and-see policy. TFT repeated after 6 weeks showed that the TSH value had risen to 61 mIU/L and FT to 4 pmol/L. He was accordingly started on levothyroxine (100 mcg, daily), and his TFT normalized after 6 weeks.
• ***Case 2:*** A 50-year-old Filipino male, with a medical history of asthma and hypertension, presented with respiratory symptoms and tested positive for COVID-19 infection. His chest X-ray showed bilateral lung infiltrates. He was treated for COVID-19 pneumonia and as per the hospital protocol (azithromycin: 500 mg daily, ceftriaxone 2 mg IV once daily, hydroxychloroquine 400 mg daily, lopinavir/ritonavir 200/50 mg bid, ribavirin, and oseltamivir 150 mg bid). His hospital course was complicated by acute respiratory distress syndrome requiring intensive care unit (ICU) admission. He accordingly received tociluzimab and methylprednisolone, but did not require endotracheal intubation or mechanical ventilation. TFTs were conducted because of persistent tachycardia, which showed TSH of 0.08 mu/L, FT3 of 3.4 pmol/L (NRR 3–6), and FT4 of 23 pmol/L. When TFT was repeated after 6 weeks, it showed resolution of the thyroid dysfunction with a TSH of 0.47 mIU/L and FT4 of 18.8 pmol/L, suggesting “thyroxine thyrotoxicosis” due to COVID-19-related thyroiditis. Subsequent follow-up over 6 months revealed no further abnormality.
• ***Case 3:*** A 32-year-old Sudanese female patient had hypothyroidism during her adolescence, for which she was treated with levothyroxine until the age of 16 years. She has been an euthyroid since then. She also presented with a history of thyroid nodules in 2016, which was not followed up later. In September 2020, she was diagnosed with COVID-19 following a mild respiratory illness. She made an uneventful recovery, but, after 3 months, in December 2020, she presented with the symptoms of tiredness, fatigue, bilateral hand tremors, palpitations, increased sweating, shortness of breath, and irregular periods. Her physical examination was consistent with a hyperthyroid state, with a small diffuse goiter. TFTs revealed TSH < 0.01 mIU/L, FT4: 72.3 pmol/L, and TRAb 28 U/L (normal < 0.75). A thyroid nuclear uptake scan with technetium revealed an enlarged thyroid, with an overall picture suggestive of diffuse Grave's disease (GD). She was accordingly started on carbimazole 30 mg, daily, along with propranolol (40 mg bid) and she improved. Repeated TFT after 2 months revealed TSH < 0.01 mIU/L, FT3: 7.6 pmol/L, and FT4: 24.0 pmol/L. Subsequent TFTs showed a normal level. She was followed up at the Endocrinology Clinic for her GD, and the latest TFTs conducted in January 2022 showed persistent remission with carbimazole.
• ***Case 4:*** A 40-year-old Filipina lady with a history of asthma on a salbutamol inhaler was admitted to the hospital with COVID-19 pneumonia of mild to moderate severity. She was accordingly started on COVID-19 treatment as per the hospital's protocol *(hydroxychloroquine, oseltamivir, and azithromycin)*. She was noted to have persistent tachycardia, and hence TFT was performed, which showed hyperthyroidism with TSH < 0.01 mIU/L, FT3 30.8 pmol/L, and FT4 83.3 pmol/L. Anti-TPO Abs; 494 IU/mL. TRAb was elevated at 8.7 IU/L. She was then started on carbimazole; however, she stopped it after her discharge believing it to be a COVID-19 medication. After 8 months, she presented to the Emergency Department with severe abdominal pain and vomiting. Her examination revealed BP: 211/110 mmHg, heart rate: 136 bpm, a small goiter, but no other signs of hyperthyroidism. Computed tomography of the abdomen showed intestinal obstruction. Laboratory results revealed TSH < 0.01 mIU/L, FT3: 32.9 pmol/L, FT4 >100 pmol/L, and TRAb elevated at 7.7 u/L. Her Bursch Wartofsky's score was 35, and hence she was treated as an impending thyroid storm. Her neck US revealed enlarged thyroid lobes with increased vascularity. The intestinal obstruction was managed conservatively. She improved and was discharged home on carbimazole (20 mg twice daily) and metoprolol (75 mg twice daily).
• ***Case 5:*** A 40-year-old man was referred to the Endocrine Clinic for hyperthyroidism. He reported symptoms of loss of taste and smell, muscle aches, and pains for 3 months; however, he was not tested for COVID-19. After 2 months, he developed symptoms of weight loss and anxiety and was accordingly referred to the endocrinology clinic. His TSH value was < 0.01 mIU/L and FT4 was 100 pmol/L. He was accordingly started on carbimazole 40 mg/day. After 2 months, his TFTs normalized. The presumptive diagnosis was GD precipitated by COVID-19.
• ***Case 6:*** A 14-year-old Qatari girl was referred to the endocrine clinic in November 2020 for possible hyperthyroidism. All family, including the patient, suffered mild COVID-19 infection and all made uneventful recovery. She complained of weight loss (around 6–8 kg) and palpitations. Her TFTs showed TSH < 0.02 mU/L and tbl4 35 pmol/L. She was given propranolol, 10 mg thrice daily. Repeat TFTs, 5 weeks later in January 2021, showed TSH of 5.6 mIU/L and FT4, 25 pmol/L. Propranolol was then stopped. Repeat TFTs on March 15, 2021, showed TSH 7.5 mIU/L and FT4 17 pmol/L. Further, TFTs confirmed euthyroidism with the complete resolution of all abnormalities.
• ***Case 7:*** A 32-year-old Filipina female with no history of any medical illness contracted COVID-19 in October 2020. She developed a mild illness. Four weeks later, she was referred by a primary physician to our Endocrine Clinic with symptoms of weight loss, palpitations, increased sweating, and shakiness. Clinically, she was in a hyperthyroid state, with TFT: TSH < 0.01 mIU/L, FT4: 46 pmol/L, TRAb: 8.7, and anti-TPO ab: 73 IU/L. US thyroid revealed bilaterally enlarged thyroid lobes with increased vascularity. She was diagnosed with GD and started on carbimazole (30 mg daily) and propranolol (40 mg twice daily). On follow-up, she remained euthyroid both clinically and biochemically.
• ***Case 8:*** A 33-year-old Filipino gentleman was diagnosed with Graves’ hyperthyroidism in September 2020, following symptoms, TSH < 0.001 mIU/L, tbl4 >100 pmol/L, tbl3 39 pmol/L, TRAb 33 IU/L, and anti-TPO Abs of 119 IU (NRR, 40 IU). He was started on carbimazole and achieved remission in a few months. In January 2021, he received radioactive iodine therapy as a definitive treatment. He remained in remission after that, and, on April 19, 2021, she got infected with COVID-19, after which a mild course requiring only simple analgesics was run. On May 3, 2021, he was examined for profound tiredness and his TFTs revealed a TSH of 90 mu/L and tbl4 < 4 pmol/L. He was started on levothyroxine (100 mcg) and his follow-up after 3 months signified the requirement for replacement therapy.
• ***Case 9:*** A 33-year-old Indian lady was diagnosed with uncomplicated COVID-19 in June 2021. In August, she was reviewed by her primary care physician for intense aches and pains along with joint discomfort. Investigation revealed only picture consistent with central hypothyroidism, with a low free tbl4 of 4.1 pmol/L and inappropriately low TSH at 6.5 mIU/L. Serial TFTs up to early December 2021 confirmed persistent central hypothyroidism, which necessitated thyroxine replacement therapy. Tests for pituitary function revealed normal results. The symptoms resolved following thyroxine treatment.
• ***Case 10:*** A 51-year-old Filipina lady with thyrotoxicosis for 15 years was referred by her primary care physician in August 2021. She was in remission 13 years ago after taking medications for 2 years. She recently contracted COVID-19 on June 17, 2021, and, after a few weeks, she noticed weight loss and eye bulging. TFTs confirmed hyperthyroidism, with TSH 0.005 mIU/L, FT3 16.3 pmol/L, FT4 52.5 pmol/L, and TRAb 9.3 IU/L. She had TFTs in November 2020, which showed normal results with TSH 0.92 mIU/L and FT4 19 pmol/L. She was accordingly started on carbimazole, which resulted in a good response with remission in the following 2 months. Latest TFTs revealed persistent remission on maintenance carbimazole.

Table 1 A summary of 10 cases of thyroid dysfunction related to COVID-19 infection discussed in the report.

## Discussion

COVID-19 infection continues to ravage the world population, and its effect proved to be diverse with multi-system consequences. Covid-induced thyroid dysfunction is emerging as a well-established and common aftermath of the disease.^4^ As demonstrated by our case reports, the clinician in endocrine practice could face diverse presentations. Five of our 10 cases presented with immune-mediated hyperthyroidism, and 4 had virus-induced destructive thyroiditis, which proved to be transient in 2 cases, and had a permanent effect in the other two. One patient had central hypothyroidism. Triggering of various autoimmune diseases in general by Covid-19 has been recognized, and includes anti-phospholipid syndrome, autoimmune thrombocytopenia, hemolytic anemia, and Guillain-Barre syndrome, among others.^14^ This may persist long after the resolution of COVID-19.

The literature shows that non-thyroidal illness (NTI) is the most common abnormality observed in COVID-19 patients. Up to 30% of COVID-19 patients may suffer the syndrome of low TSH with low or normal tbl4 and low tbl3.^15^ NTI may occur at all spectra of the illness. It commonly occurs with the “Cytokine Storm,” also known as Cytokine Release Syndrome.^16,17^ The degree of reduction of TSH and FT3 is proportional to the severity of COVID-19 infection.^18^ Those with low tbl3, generally have a low fT3/fT4 ratio ( < 0.3) suggesting a reduced 5-mono-deiodinase activity (both D1 & D2), that converts tbl4 to tbl3. We did not observe this abnormality in our case series.

Viral infection is frequently cited as an environmental trigger for autoimmune thyroid diseases.^19^ The evidence for the COVID-19 virus to amplify or promote autoimmune thyroid disorders is compelling. Several cases of GD have been reported since the zenith of the pandemic in 2020. Most cases occurred a month or two following COVID-19 infection.^20–23^ These reports were in accordance with our observations. A previous history of GD in remission was observed in most cases. Harris et al. reported a case wherein a young healthcare worker developed severe thyrotoxicosis after recovering from a mild COVID-19 infection.^24^ Jimenez-Blanco et al. reported 2 cases of GD in remission in 2014 and 2015, who presented with severe recurrence of disease more than one month after recovering from COVID-19 infection, which improved with antithyroid medications. All patients showed classical biochemical evidence and florid clinical symptoms of Graves’ hyperthyroidism, and most had positive TRAb. They were treated with antithyroid drug therapy (ATD)s and responded well. One patient^23^ had concomitant Graves’ orbitopathy at the time of the onset—this feature has not yet been reported by other reports.

On the other hand, Lania et al., in a retrospective analysis, recorded a prevalence of hyperthyroidism in 20% of a group of 287 patients hospitalized for COVID-19, with 48% of those affected reported florid hyperthyroidism.^25^ Given et al. showed that the incidence of hyperthyroidism is correlated with the severity of COVID-19 infection as it was more prevalent in those with pneumonia.^26^ Therefore, this observation may indicate the bimodal nature of hyperthyroidism triggered by COVID-19, occurring either at the time of acute illness, and mostly as transient or subclinical,^25^ or occurring on an average of 1-2 months post-COVID-19 infection, with a more protracted form of hyperthyroidism akin to Graves’ hyperthyroidism. Two of our 5 patients had a similar clinical course.

Destructive thyroiditis, which could be subclinical or clinical, may take the form of either subacute thyroiditis (SAT, De Quervain thyroiditis) or painless thyroiditis. This is well recognized to be associated with several viruses such as mumps, adenovirus, Epstein Barr virus, cytomegalovirus, influenza virus, and Hepatitis E.^27,28^ Several case reports of SAT due to COVID-19 have been published in the literature since the onset of the pandemic.^29–31^ A subacute disease occurring in 10%–20% of COVID-19 patients may occur either simultaneously with COVID-19, especially those with severe disease admitted in the ICU,^25^ or during the few weeks to months following its resolution, as noted in our patients and those reported by Brancatella et al.^32^ Neck pain is the hallmark of this disease in SAT. The classical course includes an initial phase of hyperthyroidism followed by hypothyroidism and then euthyroidism, which unfolds over 6 months. The outcome is either complete resolution^31,33^ or the development of permanent hypothyroidism.^32^ Our series included 2 patients with SAT, one of them, a young girl, recovered completely, while the second one developed permanent hypothyroidism that required replacement thyroxine therapy. A variant of subacute thyroiditis, which occurred in 15% as a form of ‘SAT’ without neck pain, is thought to be attributable to lymphopenia,^34^ which was first recognized in critically ill patients admitted to the ICU.^35^ The biochemical hallmarks comprise low TSH, low FT3, and normal or high FT4, hence the synonym “*Thyroxine thyrotoxicosis.”* This was represented in our third case with thyroiditis. Recently, Trimboli et al. published a systematic review on SAT resulting from COVID-19.^36^ They confirmed that 83% of all SAT cases occurred after COVID-19 infection, with a female preponderance, onset within 3 days to 2 months of the primary infection, and age range of 18–69 years, with complete remission in most patients, with residual hypothyroidism ensuing in only 14.8% of all patients. One of our two patients, was the youngest with SAT due to COVID-19 infection.

Primary hypothyroidism is a rare complication, occurring only in around 5% of all patients, being subclinical in 90%, and clinical in 10%.^25^ It can be chronic autoimmune thyroiditis. Another study from Iran revealed a similar prevalence.^37^ A prospective study of hospitalized patients from Hong Kong also showed that elevated TSH is a rarity among patients with COVID-19 infection and abnormal TFTs.^17^ It may occur during or after COVID-19 infection.^38^ This notion was exemplified by our first case in the series. The patient developed hypothyroidism about 2 months after contracting COVID-19. After observing for over 3 months (as he was asymptomatic), thyroid function abnormalities did not resolve, and was started on levothyroxine, following which TFT normalized. Of interest, is our 8^th^ patient who developed severe and profound hypothyroidism a few weeks after the COVID-19 infection. Despite the possibility of radioiodine-induced hypothyroidism in his case, considering the rapidity of the hypothyroidism following COVID-19 and the fact that he remained euthyroid between January and April, implies either a direct effect or an amplifying effect of the coronavirus.

Importantly, central hypothyroidism is rare, with reports of reversal of hypothyroidism in most patients. This may represent a transient effect on the HPT axis. Murugan et al. reported that 4 (6.7%) of the SARS patients 3 months following recovery were biochemically hypothyroid, including 3 with central hypothyroidism and 1 with primary hypothyroidism due to new-onset chronic lymphocytic thyroiditis.^40^ While central hypothyroidism was spontaneously remitted in the 3 patients with central hypothyroidism after 3–9 months, most patients with primary hypothyroidism generally required permanent tbl4 therapy.^40^ However, our experience with 1 patient implies that the outcome may not be predictable and that therapy can be required for managing symptoms and treating persistent biochemical hypothyroidism.

The thyroid may be indirectly damaged from hyperactivity of the Th1/Th17^40^ and the surge of “Cytokine Storm,” which possibly triggers and perpetuates the thyroid gland inflammation.^3^ Lania et al. reported an inverse relationship between TSH and the level of IL-6 in their cohort of 287 patients.^25^ This finding suggested the role of cytokines in perpetuating thyroid dysfunction. On the other hand, an extensive injury to the “follicular and parafollicular” cells caused by the virus has been reported by some studies.^41^ This result may facilitate the comprehension of the increased risk of osteonecrosis in COVID-19 patients.^42,43^

Postulated mechanisms of the trigger of Graves’ hyperthyroidism were reviewed by Murugan & Alzahrani.^40^. The possible role of COVID-19 in triggering thyroid autoimmunity is supported by several reports of thyroid dysfunction triggered by vaccines developed from these viruses^45–47^ or the worsening of pre-existing Graves following vaccination.^48^ The phenotypic expression of thyroid autoimmunity has been attributed to the balance between Th1 and Th2. A predominant Th1-mediated immune response is likely to trigger an apoptotic pathway in the thyroid follicular cells, destroying thyroid cells. Th2 immune-mediated activity is likely to activate antigen-specific B lymphocytes to make TSH-Rab, resulting in the proliferation of thyroid cells and the hyperactivity of the gland.^40^

A major limitation of this study is that it is a case series of 10 cases selected from our outpatient clinic. Detailed observation and follow-up of the patients with COVID-19 infection and thyroid illness should be conducted in the future to establish the connection suggested by the present findings.

## Conclusions

In conclusion, the evidence for COVID-19 leading to thyroid dysfunction is gradually accumulating, indicating a bimodal distribution and occurring along with an acute infection as well as during the recovery period. Our report provides further support to this notion. Thus, screening during the follow-up period is highly recommended in patients who have recovered from COVID-19 infection, especially among those with a history of thyroid autoimmunity.

## Declarations

### Ethical Approval and Consent to participate

The case series report was approved by the Medical Research center, Hamad Medical Corporation, Doha, Qatar, MRC 04-21-409. A waiver of informed consent was obtained as the report does not contain any identifiers of patients involved.

### Consent for publication

Consent for publication was approved by the Medical Research center, Hamad Medical Corporation, Doha, Qatar.

### Availability of supporting data

The data that support the findings of this study are available from Cerner at Hamad Medical Corporation but restrictions apply to the availability of these data, which were used under license for the current study, and so are not publicly available. Data are however available from the authors upon reasonable request and with permission of Hamad Medical Corporation Medical research center.

### Acknowledgments

None.

### Competing Interest

No conflict of interest is declared by the authors

### Research Funding

None

## Figures and Tables

**Table 1:** Summary of Reported Cases:

Clinical Characteristics	Age	Gender	History of Thyroid Disease	Diagnosis	Presentation Since COVID-19 Infection	Treatment Received for Thyroid dysfunction

Case 1	49	Male	No	Hypothyroidism	6 weeks	Levothyroxine

Case 2	50	Female	No	Sick Euthyroid Illness	During admission for COVID-19 Pneumonia	None

Case 3	32	Female	Hypothyroidism	Grave’s disease	3 months	Carbimazole

Case 4	40	Female	No	Grave's disease stopped antithyroid drugs prematurely and later presented with Thyroid Storm	2 weeks initially, later with thyroid storm after 8 months	Carbimazole & Propranolol

Case 5	40	Male	No	Grave's disease	2-3 months	Carbimazole & Propranolol

Case 6	14	Female	No	Thyroiditis	6 weeks	Propranolol

Case 7	32	Female	No	Grave's disease	4 weeks	Carbimazole & Propranolol

Case 8	33	Male	Yes	Hypothyroid post Radioactive Iodine ablation for Graves’ disease	2 weeks	Levothyroxine

Case 9	33	Female	No	Central Hypothyroidism	2 months	Levothyroxine

Case 10	51	Female	Yes	Grave’s disease	2 months	Carbimazole

